# Supplementary material for: Comparative Genomics of Staphylococcus Reveals Determinants of Speciation and Diversification of Antimicrobial Defense
Source: Front Microbiol. 2018 Nov 19;9:2753. doi: 10.3389/fmicb.2018.02753 (PMC6252332; doi:10.3389/fmicb.2018.02753)
Supplement: FILE S1 — Staphylococcus species and strains used as substitutes in OrthoMCL analyses. [file Data_Sheet_1.PDF]

Table S1

**Species**	**Strain**	**Accession**	**Status**
**Group A**			
S. aureus	USA300	CP000255 (Uid58555)	Complete Diep *et al*., 2006.
S. epidermidis	RP62A	CP000029 (Uid57663)	Complete Gill *et al*., 2005.
S. hominis	J11	LT963438	Draft (This study)
**Group B**			
S. saprophyticus	CCM_833	LT963436	Draft (This study)
**Group C**			
S. pseudintermedius	ED99	Uid162109	Complete Zakour *et al*., 2011

Diep BA, Gill SR, Chang RF, Phan TH, Chen JH, Davidson MG, Lin F, Lin J, Carleton HA, Mongodin EF, Sensabaugh GF, Perdreau-Remington F. 2006. Complete genome sequence of USA300, an epidemic clone of community-acquired meticillin-resistant *Staphylococcus aureus*. Lancet. **367**:731-739.

Gill SR, Fouts DE, Archer GL, Mongodin EF, Deboy RT, Ravel J, Paulsen IT, Kolonay JF, Brinkac L, Beanan M, Dodson RJ, Daugherty SC, Madupu R, Angiuoli SV, Durkin AS, Haft DH, Vamathevan J, Khouri H, Utterback T, Lee C, Dimitrov G, Jiang L, Qin H, Weidman J, Tran K, Kang K, Hance IR, Nelson KE, Fraser CM. 2005. Insights on Evolution of Virulence and Resistance from the Complete Genome Analysis of an Early Methicillin-Resistant *Staphylococcus aureus* Strain and a Biofilm-Producing Methicillin-Resistant *Staphylococcus epidermidis* Strain. J Bacteriol **187**:2426-2438.

Zakour NLB, Bannoehr J, van den Broek AHM, Thoday KL, Fitzgerald JR. 2011. Complete Genome Sequence of the Canine Pathogen *Staphylococcus pseudintermedius*. J Bacteriol **193**:2363-2364.
