# Supplementary material for: Comparative Genomics of Staphylococcus Reveals Determinants of Speciation and Diversification of Antimicrobial Defense
Source: Front Microbiol. 2018 Nov 19;9:2753. doi: 10.3389/fmicb.2018.02753 (PMC6252332; doi:10.3389/fmicb.2018.02753)
Supplement: FILE S2 — Species group A, present and absent cluster protein sequences. Sequences represent clusters listed in Table 2. [file Data_Sheet_2.PDF]

Group A formation proteins from randomForest analysis

>epi_00394
MSFNEIIFKNFKQNVTHYAIYLLSLVISVVLYFSFVTLKYAHHL
HGNQSFPAIKEGSQVGSYFLFIIIVVFLLYANFLFLKRRGRELSLLQIIGLTKKDIMK
MIMLEQLMTFMMTTIVGIILGIFGSKILLMIVLRLLGINVSVSIIFNYHAILETLLLI
AVSYVLIVFQSYVYLLKRSIKELASDVNKKEFSHTRTTLGEVVLGLLGIIMIVSGYIM
STKLVDNVETIIQPFAILFLTVIGSYFFFRSTVSLIFKAIQRLRNGTVSVTDVMFTSP
IIYRVKKNAFSLTVMTVVSAITVSVLCFAALSRSTLTNEVLLSSPHDVTLKNQKQANE
LAFKLNNRNIEHYYNYKEVVYAKVYKDHLFSEGVYRPKEITVTSDKYIPNVSTKKGQT
DIIIPRGSLKDVVKTDKKRTAYVGSKKFRLKVSLRKGINKVYFMSDVDRGRPTLILND
EDYQKIREHIKEKNIVSQYGFDLKNKNDLPELEKLVSSINEDIETRSEAASEISSLTG
ILLFVTSFLGITFLIAAGCIIYIKQIDETEDELENYTILRKLGFTHQDMSKGLKLKVI
FNFGLPLIIALLHAYFASLAYMHLMNVTNQIPIFIVMAVYTATYAVFAIIAYNHSKRT
IKHSI
>epi_00430
MKNLFLSSVAVILSICLVLLLFLATNQNALAKVHKTICTFTKVK
TVSHNDIFPNLNMNHVAEASEKNSDHQKNVNKTNQQSVRTQASYDFNPQECKVIALNY
AENHLSKKYNIERTFNESLHSVYVFSSENKLHQTKVKVNKSGTVHASNINNH
>epi_00538
MKDWTINNHSKRQLIIQSNHDDISIIEPLRNGSFKILAEFNALN
TDYIQVYDHTLVVSIHEKEISVFDRSSQSI
>epi_00542
MKLISKSIYLSFIFFIFSLMLFTNQSFANDNKRFQLKPGDIIVT
KGPVLNGFFGHCSLAIDHDTVLQIEGPGDKPMTEDFESYRDRYGKGKNEWIKVYRCSH
PNAGTKAAQWAKKHYENSDSEYLVTFNLKSETFTYCTKIIYQAYKYGVDKNSVSDHGL
YIISPYALTDNFTDEYKLKLVKTY
>epi_01643
MKNQDKIQNVVKLLSSLGVNIKKTKSRLDIINTLPTSNQVSHELK
>epi_01831
MQQQKIKRFLAHFVLDHVTIEYLKYGHQLTIEQLKLLIFILHFT
ENHKEDLSLNMIIFYKNYQKNQLLKSITHLYEFNWISKKRHPYDQRRLVITLTQNQCS
KITQLIEELEHFLEVKSTLINEINHSTLLSYYLKCHSQFRVIEQSCTSQHLTLEELYL
LGLLIISDNKTTFKSIKVHALKGIIAMGPIIKTLQSKGYLIKSRSRDDERYIVLTLRK
EKVNVIQSEIEECYNKLEQGIQHV
>epi_01832
MRENYSIRVFNNLAINTEKALSVKVFYCLKGKCEVTINVQKHIL
EKDDIAFVVMNDTYSLLSNTETMCCIIDIPIHRYLSQKDTQFLISGTKIDESSRDRIK
YWILKILELHCLSYNDVSEIQRLIQFLLIELSYLKKPKLDVNKDFYLSEDIHQYLVDH
HDSKINKHELAEAVNLSNQALTSMFKQTPFQTFNQYLNQLRLKFCLIDILTTHKPIEE
IAIDHGFHHYSRFIQLFKNTYGYTPKLIRRDYIATSIFKNTAEEIDLDRHFLMNIHEL
QDLDSKIISKKYIKMSDKGKKYRSYDIYIEDNRSTILDQEQIVHIKRNLSLSQKSMRY
VIELNYTSMIENKELCRYEMLKILRFCSGLNLIPTFKIITDRHDTFTSKEKMALKLTF
QMLFIMLREFNQLEIEFIVEDMMLKQVVQLKKMISSYFEYYKLNYRIKNEKIGNVNYQ
NLEKQVTQIFIPIDQLHLYIKEMSFEKVILETSYLTDLKSEVVEYPWLQHIHTLIKMC
GLVRGVLIQPSFDYATTYTYHSNLKPHHILSYVIRMFNQLRGTIVYKDDAIIMTKYKY
EYQAIAIFLKNRIKENVNHQQLIFSDIQSLNHAEYEYIEMMPLLMNENNELDSHIPYP
HQYWLAKLKSKESQQAIVDLPKMSIAHLTFLCS
>epi_01980
MDIKQSSEKQGRPHHLSDSRTVLKRNFILIPTYILLQSIIPIIV
VFGSLGITAMITQQAPPQWLYHCSLSLSFVIAQGLILIIFYKIHQSVINDVMKQQWII
AKNKIIKIVIVAIVVYLLLLIIRVIGTSLPNHLSYHLTQSEQRTLGLFKSPYVLLVTF
ISMVFLRPMVEQIIYRYLIIHELGKVWNRQFVIGLSIVIETIVHVYDMASIFEIFPYI
VIASAATILYIKSRDNLIVAYIFQVILQCILFIEILCKYTNF
>epi_02055
MSFYEFMQNFLGDDTPLGELVDWINQDINFPKDVTSQKEILTYF
RNHPCPENIPVTSIKRALAVFNQFTKV

>epi_02098
MKKISLIATTVLTGLLLFPSVNDTTTHAAEVTSHDAQAVAIQAM
KNSGGNPDLQNFKKVKDKGDYFTIDINNKSGAGVGTYKVYKNGVVLYKSGNYGEYSQL
NTRQCYVAQDIATTSSQVKRHSTQQTQAVDSTRELNSYYVGQVQSSDLPQQSSSDMLP
NTGMKDKNFNTNGIISLFLLTAGFITLYHQPLRKMS
>epi_02108
MSFYHFIQNFVGDETPLGKLATCINQDEDLPMEETTAHNILNYF
NQLNYFDDDCIEAVKRSLSLYEQSKVAL
>epi_02134
MTFKILWKMIKQNFVNQRHIIVPFISVISILFGIEYILLSLTTN
IYFNEHHPELKISAIIGIVFMTMLLFIFLIYANHFVMNHRKKEFALNMALGMEKKHLR
LIILIELFIQFIISAILSIVGGYLFGELFFMLFNKIVNTHQPQLSDYPFDVLSMKITL
TMLLSLMIILFVINNFKISFKNSLQLLIKNKSKTHEKSRVRLIIFLILGLIFIGIGYY
LAIKPNTAIGSLGIIFFAILSTLIGTYLLFVSLGSIVLEMLQKLDHYYYKPNHFFFIA
GLKSRVKSSAIGLATISFMCTFLIVTLSMTVSTYRNMDHRFEFAFKNDYAGYYIGDFH
KDSKIQRKIENLKKDIRQEVPTGQFKIYARGMVGAELQGGLKHKKLKRQTVSSGLFNF
GNKQKFNSFISIYNKSDYNKNNKKIKLDDDEIAISTSVSLFKKMKTLNIFGKTYRVKY
IESTNIDNLLYADGITLIVNQQQLMDRIVNEYRNHNDENLIITPNQVQTAVEFNVLKE
KDKLNHRIKKIGVQHDIEFQVKKQNLLMWKQVNSSLIFVGSVVSLVLLIGIFLMMYYK
QVSEGHEDRDAYITMKQLGLDEILIKKTINKQVIWVFLIPVIVAIIHTLAAFRIIYSV
LGIVGQYDLGLYATSYVGVIVVFIIFYSMMYWITSRIYYTMINDKH
>epi_02135
MLLEVKNVKKVYGRGMNTTMALNQMNLEIDENEFVAIMGESGSG
KSTLLNLIATFDRTTEGLIKLDGLPLNQLKNKDIARFRREMMGFVFQDFNVLNTMSNK
DNILMPLVLANERPKIMQKRLMEISEQLGIEDLLEKYPSEISGGQKQRIAIARALIAR
PKLLLADEPTGALDSKTSKNLMCLFRKINQKHQTILMVTHSNIDASYANRVIFIKDGR
LYHEIYRGEESQTDYQKRIADSLAILNGVGD
>epi_02136
MKFAYIQSIRNEISIILIIILLFFALIFYVFSLPFDAYVLAISII
LLLMCVRWWIKYLSFKKNEHLKDKVAYLEHELAHVKNQQIEYRNDVESYFLTWVHQIK
TPITASQLLLERNEENVVNRVRQEIVHIDNYTSLALSYLKLLNEESDMTITKVTVDDL
IRPLILKYRIQFIEQKTQIHYEKSEDIILTDAQWASIMIEQLLNNALKYAKGKDIWID
FDVANQTLQIKDNGIGISKADIPKIFDKGYSGFNGRLNEQSTGIGLFIVQHIANHLNI
QVTVQSELNHGTVFFIHFTKEK
>epi_02137
MKIFIVEDDLVIAESLANELSKWNYEVHVVDNFEKIMEDFRRVE
PQLVLLDINLPTLNGFHWCQEMRKVSHVPIMFISSRTDNMDQILAIQMGGDDFIEKPF
NLSLTVAKIQALLRRTYDLSIARDEIAVKGCKLIVDEAKLSKDNEHVQLSLTELQILK
LLFQNENKYVSRTALIEKCWESENFIDDNTLAVNMTRLRKKLLSIGVDDLIETKKNVG
YRV
>sap_00201
MTKLEVINPATNEVLERLDYATHEQINHQIKQAHQAFQNWKKVDAHERSAKLAQWAQLID
DHQDELARLITLEGGKPLAEAKGEVAYANSYVKWYAEEAKRVYGRTIPANSPSKKIVIDK
FPVGVVGAITPWNFPAAMITRKMAPALAAGCTIICKPAVKTPLTTIKLVELAHQAGFPKD
AISYIIASGKDAGDIFTNHSLISKVTFTGSTAVGKSLIESSAQQVKNVTMELGGLAPLIV
HNDADIEAAVDGTIASKFRNAGQTCICANRIYVHEDIAEAYNEKLIEKVHALSVGDGLKE
EVKIGPLIDNQAVEKVLTHIKDAQEKGGQLSRSIEDIQALGGNFLKPVVITNANLDMKAM
HEETFGPVAPVMTYSDLDEVINIANDTEFGLASYFFTNDYRTGLKLFNELEYGVIGWNDG
GPSAAHAPFGGLKESGYGREGGVEGIEPYLETKYLSIQE
>sap_00203
MKNHLHRWVIEALSFMALGLFGSLIIGLILETIGNQTLIPQIDLSFLSEIGKVAMSVTGA
AIGVAIAYGLGAKPLVIFSCVIVGTLGYTTFGGGAVGAYIATLIVVELSRFYASKTKIDI
IVTPLLTIVIGGAIAKLFGPALNEFMTGLGKVIIVATEQQPLIMGILVSVLFGLALTAPI
SSAALALMLDLSGLAAGAATIGCAAQMVGFAVNGYKDNGWSGILSIGIGTSMLQVPNIIK
HPMILVPPTIASAVVAPVMTTLFPMTNNAAGAGMGTSGLIGQIMTINTMGGSLQTWLLII
IFHIILPAVISFALYKYFFKKQWIMPGDQLIHVAN
>sap_00398
MKVFLKLGWFFKQQKTSYLIGLGTLLLIALIEIVPPQIIGRTIDEMTSNRLTPRLLAIYL

FILVITAILTYVLRYVWRLSIFGTSQKLGKILRTYLYKKYTEMSAIFFQNRRTGDLMAHA
TNDIRAVQNAAGAGILMIADSLITGGTVIITMATTVSWKLTLIAMIPLPFMVLLTSIYGS
LLSKGFKKAQAAFSKLNDKTQESVAGIKVTKTFGYEPSDQADFKHLSDDVVAKNLKVSKI
DALFDPTITLVIGMSYFLSIAFGAQMVFHNDISLGQLITFNTYLGMLVWPLLALGLFFNI
VQRAKASYERIEEIGELPNDIDTSYVIDERPQGDIRFNINQFYFPGNEDQGIYDIHFTIK
EGSTVGIVGRTGSGKSALIRLLLREFDTQHAQDIEFGGHPIRDYNVESLRAQFGYVPQEH
FLFSTTIRNNIAFSNETIDDEKIFEASKLSHIHDDIMQFSKDYQTVVGERGVSLSGGQKQ
RVSIARALLVNPEILILDDSLSAVDAQTEEAILENLERLRSGKTNIITAHRMSAVKHADL
IIVMNEGRIIERGNHATLMSKKGWYYDTYQAQALQEQLSRNLDSLTKGDGEND

>sap_00399

MTENANLTAKDQGSALIRLFKYTLPYKWIIVLAFITLILSTIASMMTPYMVKIFIDDYLT
PRHFPKETMVWLIVIFISIQLIGAITLYFSQYLFQYLAFKVIQQLRIDAFNKLGKLGMRY
FDKVPGGSIVSRLTNDTETIVDMIVGVFSTFIMAFFMMISSYIMMFVLDVKLALIALIFL
PIIMIILASYRKYSAFLFSKSRQRLSDLNSKLAESIEGMKIIQAFNQERRLNKEFNKIND
EHYQYMLKTVKLDSLLLRPAISSISIFAVVMILGYFGVISFTTGITAGVVFAFVQYMERF
FEPINQVSQNLNILQQALVSASRVFALINDDTYEPQQEANNDNAIETGEIEFDNVSFSYD
GETDVLKNISLTAKPGEMIALVGHTGSGKSSIINLFMRFYEFNRGDIKIDGNSIKKIPKT
ELKEKIGLVLQDAFMFYGTIASNIKLYHPSMTFEQVKAAAEFVHANHFIEKLPNQYQHKV
IEKGSAFSSGERQLIAFARTIATNPKILILDEATANIDSETEEQIQQSLNKMRKGRTTLA
IAHRLSTIQDADQIFVLNKGEIVERGTHAQLIAQKGIYHNMYLLQNG

>sap_00643

MSVLVLGGAGYIGSHAVDQLISRGYDVVVVDNLGTGHRAAVNKDARFYEGDIRDKPFLDQ
VFTTETIEGVFHFCAYSLVGESVEKPLAYFNNNVNGLQVLLEVMYDHQVKHIIFSSTAAV
YGEPDTVPITEADSKTPTSPYGESKLMMEKMMHWSHNAYGVNYAALRYFNVAGAKEEGTI
GEDHRPETHLVPIVLQVALGQREALTIFGDDYDTEDGSCIRDYLHVVDLIDAHILAYQHL
QNGGESGAFNLGSSQGYSVFEILEAARKVTNKPIEAKVGPRRAGDPSKLVASSDKAQSIL
GWKPKHDNIHDIIDSAWRWHNQHPNGYQEDTEV

>sap_00760

MNISNPKIYEQVADIILENIKSEDYQVGDKLPSIQKLSKQFGVSTASVREALNALRTIGV
IEMKQGYGTFIKQIEPTFFEFGDKFNSLVQIKELLELREIVESATVANAARYRNKADLEK
LATALSVMGEAVIDGTSGEKADLEFHLAIAHAAQNSLLVELLNNISELMQNSMEETRKIF
IYDKQKTMTKLLEEHEMIYHAILEQDDKTAVKAMQSHLLEVKQTILANFKET

>sap_01003

MKTKHLSLKIVIALVLGIAIGSIFNVFSNTAFVENVDKYLFNVIGQIFLNLIFMLVVPVV
FVSIVLGVVGVGDPKLLGGIGIKTITFFLTTTAIAIIIGITLALVLQPGAGKSELLNSDD
VSSYQQTLEKEDSSQDSAAKQTFDQTLINLFPKNPLQAMTDENMLQIISFAIFIGVGIIM
VGSKAQMVHKFLEQTNDVLMYIVTMIMSVFAPIGTFGLVAHAFTGAGFGAIQQLGMYFFI
VLLGLGIHFFVVYGAAVKFMAKSSPLKFFKDFIPAITLGFSASSSTATLPVSLECTKKMG
VRREIASFVQPLGATINMDGTAIMQGVATIFIAQISGVTLSVSQIITVVVIAVVASIGTA
GVPGVGLIMLAMVLNAVGLDPAAIGIILGIDRLLDMTRTSGNITGDAACALVISNAEERK
LAKERTLNE

>sap_01607

MKYKTVFDIIGPTMVGPSSSHTAGAVRIGLVARDLFNQLPKQADIYLYGSFMETYKGHGT
DVALVGGLLGYDTDDDRIQTSLETAEEAGMKVNFIEMAEERSHPNTAIINMRDGDKEISV
EGVSIGGGKIEVVAINGFNIAISGNYPALLVFHKDTFGTIGRVANILGDSSINVGSMQVS
RKEKGDQALMTCELDDAVNDETIEKIKNVDGVVTVSLMGDA

>sap_02162

MKYKTVFDIIGPTMVGPSSSHTAGAVRIGLVARDLFNQLPKQADIYLYGSFMETYKGHGT
DVALVGGLLGYDTDDDRIQTSLETAEEAGMKVNFIEMAEERSHPNTAIINMRDGDKEISV
EGVSIGGGKIEVVAINGFNIAISGNYPALLVFHKDTFGTIGRVANILGDSSINVGSMQVS
RKEKGDQALMTCELDDAVNDETIEKIKNVDGVVTVSLMGDA
