# Supplementary material for: Comparative Genomics of Staphylococcus Reveals Determinants of Speciation and Diversification of Antimicrobial Defense
Source: Front Microbiol. 2018 Nov 19;9:2753. doi: 10.3389/fmicb.2018.02753 (PMC6252332; doi:10.3389/fmicb.2018.02753)
Supplement: FILE S3 — Data from BLASTp search analysis for GraS and BraS homologues in 49 Staphylococcus species genomes. BLASTp was performed using default settings and either S. aureus N315 SA0615 (GraS) or SA2417 (BraS) protein sequences. [file Data_Sheet_3.pdf]

BLASTp output of search with *S. aureus* N315 BraS and GraS proteins

Staphylococcus sp.	BLASTp with *S. aureus* N315 SA2417 encoding BraS - 295aa			Representative sequence match id and number of matches with identical output scores	BLASTp with *S. aureus* N315 SA0615 encoding GraS - 346aa			Representative sequence match id and number of matches with identical output scores
	coverage (%)	match (%)	length (aa)		coverage (%)	match (%)	length (aa)	
S. agnetis	93%	51%	285	KFE42010.1; WP_107390376.1; PTH58982.1; WP_051879704.1 (4 totral)	braS only			
S. argenteus	100%	88%	295	WP_001061266.1;CCE58461.1; EYG93235.1; EYL87994.1 (90 total)	100%	92%	346	WP_001061266.1;CCE58461.1; EYG93235.1; EYL87994.1 (90 total)
S. arlettae	graS only				98%	49%	343	WP_002508952.1; EJY96791.1; PNZ54955.1; PTH22285.1 (12 total)
S. auricularis	92%	58%	299	PNZ66384.1; WP_059107059.1; SQJ17431.1 (3 total)	braS only			
S. capitis	100%	65%	295	WP_107360978.1; WP_002454129.1; EFS16395.1; PNZ78422.1 (5 total)	100%	73%	346	WP_002469826.1; EGS39757.1; CDI72433.1; CRN11888.1 (12 total)
S. caprae	100%	66%	298	WP_103212398.1; POA07172.1; SUL94511.1; WP_002444546.1 (9 total)	100%	72%	346	WP_002441353.1; EES40977.1; BBD90781.1; BBD93271.1 (5 total)
S. carnosus	graS only				100%	51%	347	WP_015899561.1; CAL27216.1; SUL91371.1 (8 total)
S. chromogenes	99%	50%	293	WP_107369081.1; PTF75457.1; PTG53315.1; WP_107375807.1 (4 total)	braS only			
S. cohnii	graS only				98%	61%	341	WP_103211088.1; PNZ47664.1; SUM09396.1; WP_019468169.1 (4 total)
S. condimenti	graS only				100%	52%	347	WP_047130882.1; APR61750.1; PNZ63726.1 (3 total)
S. delphini	graS only				98%	49%	340	WP_096555250.1; PCF47683.1; PCF74925.1; WP_096661915.1 (6 total)
S. devriesei	98%	60%	298	WP_107507133.1; PTE69419.1; WP_107506116.1;PTE73063.1 (4 total)	100%	68%	346	WP_107507133.1; PTE69419.1; WP_107506116.1;PTE73063.1 (4 total)
S. edaphicus	graS only				98%	60%	342	WP_099089513.1; PHK50435.1 (2 total)
S. epidermidis	99%	62%	298	WP_049387329.1; WP_075778256.1; OLS07248.1; WP_088922888.1 (10 total)	100%	73%	346	WP_037552000.1; OLS04512.1; WP_032605424.1; WP_049381230.1 (116 total)
S. equorum	graS only				98%	59%	342	WP_065337469.1; WP_069815226.1; WP_069832654.1; WP_065367335.1 (20 total)
S. felis	graS only				98%	46%	340	WP_103209351.1; PNZ35255.1; AVP35693.1; (3 total)
S. fleurettii	graS only				94%	44%	353	WP_078357080.1; OOV77763.1; WP_107509169.1; PTE34731.1 (4 total)
S. gallinarum	graS only				98%	58%	340	WP_107589947.1; PTL08556.1; PTL09231.1; WP_107580330.1 (5 total)
S. haemolyticus	98%	63%	298	WP_107611263.1; WP_053036308.1; WP_053041283.1; WP_037551505.1 (8 tota	99%	67%	346	WP_037548330.1; KGF28920.1; PNN21754.1; WP_107611956.1 (14 total)
S. hominis	99%	60%	298	WP_048761836.1; WP_065581106.1; WP_017175465.1; WP_064206740.1 (8 tota	98%	65%	346	WP_107622816.1; WP_087436602.1; P_071860083.1; WP_002449136.1 (11 total)
S. hyicus	98%	51%	303	WP_107642911.1; WP_039643664.1; WP_107633684.1; AJC95003.1 (5 total)	braS only			
S. intermedius	graS only				97%	48%	340	WP_086428347.1; WP_019168595.1;PCF65534.1; PCF81212.1 (5 total)
S. kloosii	graS only				98%	56%	344	WP_103295018.1; PNZ06991.1; AVQ36881.1; SUM49981.1 (4 total)
S. lentus	graS only				93%	45%	335	WP_016998476.1; OAO21057.1; OAO29099.1; SCU24843.1 (6 total)
S. lugdunensis	98%	59%	302	WP_037545648.1; KAK55469.1; AMG64126.1; ARJ13005.1 (5 total)	100	67%	346	WP_070864798.1; ARJ19575.1; WP_002459808.1; ADC88252.1 (24 total)
S. lutrae	graS only				97%	48%	345	WP_085237678.1; ARJ51206.1; PNZ39451.1 (3 total)
S. massiliensis	98%	49%	300	WP_009382395.1; EKU50143.1; POA00837.1; (3 total)	braS only			
S. microti	graS only				98%	47%	340	WP_044360321.1; KIX90677.1; PNZ81762.1; SUM56744.1 (4 total)
S. muscae	graS only				98%	46%	339	WP_095115436.1; SNV99678.1; PNZ00910.1; AVQ34244.1 (4 total)
S. nepalensis	graS only				98%	59%	341	WP_096810771.1; ATH65922.1; PTK58769.1; AWI45311.1 (5 total)
S. pasteuri	100%	61%	298	WP_023374225.1; AGZ25449.1; WP_002467359.1; PAK72619.1 (7 total)	100%	74%	346	WP_023374745.1; AGZ26042.1; WP_015365319.1; PAK73248.1; (12 total)
S. petrasii	98%	63%	298	WP_103297242.1; PNZ31987.1; SUM42862.1; WP_103328646.1 (4 total)	100%	68%	346	WP_103365973.1; PNZ83171.1; SUM60742.1; WP_103328564.1 (5 total)
S. pettenkoferi	99%	58%	300	WP_049405395.1; WP_103369869.1; PNZ91056.1; WP_002472754.1 (7 total)	braS only			
S. piscifermentans	graS only				96%	51%	345	WP_095106673.1; SNV08337.1 (2 total)
S. pseudintermedius	graS only				100%	46%	340	WP_096548143.1; PCF67316.1; WP_101457656.1; POF44581.1 (60 total)
S. rostri	graS only				98%	46%	340	WP_103358113.1; PNZ27548.1 (2 total)
S. saccharolyticus	100%	65	298	WP_002454129.1; WP_037550651.1; WP_002444546.1; WP_115312593.1 (7 tota	100%	73%	346	WP_049428823.1; PAK62513.1; WP_002452657.1; PAK56765.1 (6 total)
S. saprophyticus	graS only			* SUM68412.1 reports a braS gene out of >25 genomes (variable or strain id?)	98%	65%	346	WP_115345247.1; SUM73963.1; WP_069994319.1; SCS40694.1 (5 total)
S. schleiferi	graS only			* SUN32591.1 reports a braS gene out of >25 genomes (variable or strain id?)	98%	47%	340	WP_050331677.1; AKS69936.1; AKS72055.1; AKS74342.1 (4 total)
S. schweitzeri	100%	89%	295	WP_047450013.1; CDR26213.1; WP_047426816.1; CDR61393.1 (6 total)	100%	91%	346	WP_047427551.1; CDR26565.1; WP_047449751.1; CDR67110.1 (4 total)
S. sciuri	graS only				96%	45%	353	WP_107565401.1; ARB40023.1; PTJ44886.1; PTJ51641.1; WP_107577114.1 (6 total)
S. simiae	98%	73%	296	WP_002464974.1; EHJ07012.1; SNV84231.1; PNZ10070.1 (4 total)	100%	81%	346	WP_002463954.1; EHJ07899.1; SNV63500.1; PNZ12188.1 (4 total)
S. simulans	graS only				100%	54%	347	WP_105993472.1; WP_107605197.1; WP_107539804.1; WP_105966405.1 (18 total)
S. stepanovicii	graS only				93%	42%	335	WP_095086018.1; SNV58008.1; PNZ75708.1 (3 total)
S. succinus	graS only				98%	57%	340	WP_101116891.1; WP_069823522.1; WP_103268112.1; WP_107553929.1 (7 total)
S. vitulinus	graS only				94%	46%	353	WP_103323259.1; PNZ36363.1; P_107536430.1; PTI29166.1 (5 total)
S. warneri	100%	64%	298	WP_037550651.1; WP_044466397.1; WP_049423181.1; WP_075778256.1 (10 tot	100%	74%	346	WP_015365319.1; WP_058709807.1; AGC91322.1; KKI61249.1 (14 total)
S. xylosus	graS only				98%	59%	341	WP_096810771.1; WP_039069321.1; SUM96500.1; OEK87946.1 (7 total)
